# Augmented visual feedback of movement performance to enhance walking recovery after stroke: study protocol for a pilot randomised controlled trial

**DOI:** 10.1186/1745-6215-13-163

**Published:** 2012-09-11

**Authors:** Heather Thikey, Madeleine Grealy, Frederike van Wijck, Mark Barber, Philip Rowe

**Affiliations:** 1 Bioengineering Department, University of Strathclyde, Wolfson Building, 106 Rottenrow East, Glasgow, UK; 2 School of Psychological Sciences and Health, University of Strathclyde, Graham Hills Building, 40 George Street, Glasgow, UK; 3 Institute for Applied Health Research and School of Health and Life Sciences, Glasgow Caledonian University, Cowcaddens Road, Glasgow, UK; 4 NHS Lanarkshire Stroke MCN, Monklands Hospital, Monkscourt Avenue, Airdrie, UK

**Keywords:** Stroke, Gait, Rehabilitation, Visual feedback, Biomechanics

## Abstract

**Background:**

Increasing evidence suggests that use of augmented visual feedback could be a useful approach to stroke rehabilitation. In current clinical practice, visual feedback of movement performance is often limited to the use of mirrors or video. However, neither approach is optimal since cognitive and self-image issues can distract or distress patients and their movement can be obscured by clothing or limited viewpoints. Three-dimensional motion capture has the potential to provide accurate kinematic data required for objective assessment and feedback in the clinical environment. However, such data are currently presented in numerical or graphical format, which is often impractical in a clinical setting. Our hypothesis is that presenting this kinematic data using bespoke visualisation software, which is tailored for gait rehabilitation after stroke, will provide a means whereby feedback of movement performance can be communicated in a more meaningful way to patients. This will result in increased patient understanding of their rehabilitation and will enable progress to be tracked in a more accessible way.

**Methods:**

The hypothesis will be assessed using an exploratory (phase II) randomised controlled trial. Stroke survivors eligible for this trial will be in the subacute stage of stroke and have impaired walking ability (Functional Ambulation Classification of 1 or more). Participants (*n* = 45) will be randomised into three groups to compare the use of the visualisation software during overground physical therapy gait training against an intensity-matched and attention-matched placebo group and a usual care control group. The primary outcome measure will be walking speed. Secondary measures will be Functional Ambulation Category, Timed Up and Go, Rivermead Visual Gait Assessment, Stroke Impact Scale-16 and spatiotemporal parameters associated with walking. Additional qualitative measures will be used to assess the participant’s experience of the visual feedback provided in the study.

**Discussion:**

Results from the trial will explore whether the early provision of visual feedback of biomechanical movement performance during gait rehabilitation demonstrates improved mobility outcomes after stroke and increased patient understanding of their rehabilitation.

**Trial registration:**

Current Controlled Trials ISRCTN79005974

## Background

Stroke is the largest cause of complex disability in adults in the UK [1] and the third most common cause of death worldwide [2]. Two-thirds of stroke survivors encounter problems in walking after stroke, with 30% still unable to walk without assistance 6 months later [3,4]. Regaining walking function is a priority for stroke survivors since this widely influences their status of independence and thus quality of life [5]. Overground gait training is a physical therapy intervention in which a physiotherapist will observe, cue and facilitate a patient’s walking pattern. It is commonly supplemented by practising walking and exercises purposely aimed at improving gait performance [4]. Stroke survivors will often employ abnormal movement patterns to compensate for reduced range of motion, strength and control. For example, a typical characteristic of hemiplegic gait is hitching of the hip when stepping forward to compensate for reduced hip and knee flexion. Patients are often unaware of their compensatory movements, and physiotherapists will aim to discourage these at an early stage by encouraging awareness of the position and orientation of their limb segments.

Feedback of performance plays a central role in skill acquisition. After a stroke, intrinsic feedback mechanisms are often impaired and so extrinsic (or augmented) feedback is of great importance for motor relearning. While there is plentiful literature to provide guidance in the use of extrinsic feedback to promote motor learning in the healthy population, more research is required to inform the use of extrinsic feedback in stroke rehabilitation [6,7]. Physiotherapists currently provide feedback in various forms, including verbal comments and demonstration. In clinical practice, physiotherapists may also use mirrors or video-recordings to provide patients with visual feedback of their movement performance. These allow patients to become aware of how they move and correct any compensatory strategies. However, these methods are not optimal after a stroke due to cognitive and self-image issues. Viewpoints are often limited using these techniques and fail to capture aspects of movement sequences that would benefit from observation in different planes. The ability to process information after a stroke can be impaired, and mirror or video-recordings present visual feedback within a relatively complicated visual field: patients can be distracted by the clinic background or distressed by their altered appearance as a result of their stroke; and the ability to observe subtle movements can be obscured by clothing. Moreover, mirrors provide visual feedback as a reflected image, which can cause difficulties for stroke survivors who find it hard to differentiate left from right.

A recent review by Laver and colleagues suggested that the use of virtual reality could be a useful approach to stroke rehabilitation [8]. However, definitive conclusions could not be drawn due to the small number and sizes of studies reviewed and the diversity of virtual reality applications (from commercial games to bespoke rehabilitation programmes). Virtual reality has the potential to increase patient motivation and engagement in their rehabilitation. Many of these applications are based on motivational games that encourage patients to move through repetitive movements. However, although these games may give a representation of the movement abilities of a patient, they tend not to show how patients move in context; that is, how limb segments move in relation to each other. This project aims to use a virtual reality platform to show stroke patients how they move during gait re-education, whilst removing distractions associated with mirrors or video feedback.

**Figure 1:**
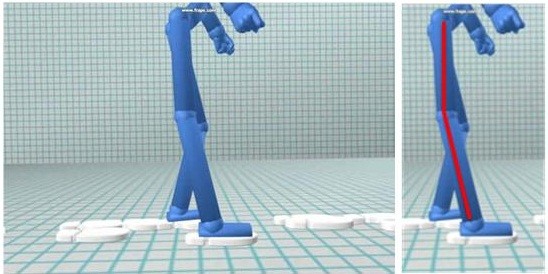
A screenshot of the visualisation software showing gait performance.

**Figure 2:**
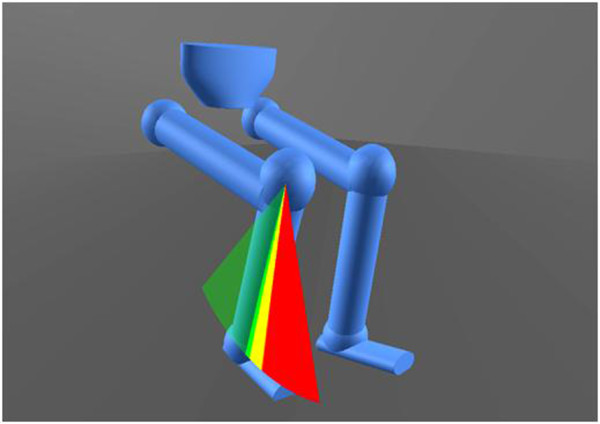
A screenshot of a virtual, gait-related target exercise in the visualisation software.

This study proposes to investigate a novel intervention strategy that uses dynamic visualisations of the patient’s movements to facilitate gait re-education. Movement data will be captured in real-time using a three-dimensional (3D) optical motion analysis system. The software package will extract limb segment position and orientation data of the pelvis and lower limbs and present this numerical data using bespoke visualisations. The visualisations take the form of a stick figure representation of the patient moving, which is shown to them on a monitor. The stick figure mimics the user either in real time or after they have completed the action depending on the task and the patient’s ability to process information. In this way stroke patients, and their physiotherapists, will be provided with immediate, clear and depersonalised visual feedback of their biomechanical movement performance. Patients and their therapists will be able to review gait performance (see Figure1), participate in virtual gait-related target exercises (see Figure2) and track progress in a more tangible way. The software will be capable of showing users how they move from different viewpoints, and will allow users to zoom in and out and have speed control over replays of their actions, whilst retaining the accuracy and numerical data from 3D motion capture.

This study is part of a wider project called envisage. The envisage project is a multicentre project with a principal aim of promoting functional independence through dynamic visualisation of biomechanical data across a range of rehabilitation settings. The collaborators are the University of Strathclyde, The Glasgow School of Art, and Glasgow Caledonian University. The project is being funded by the Medical Research Council’s Lifelong Health and Wellbeing initiative (grant number G0900583, ID 91021).

### Primary research question

Will the early provision of augmented visual feedback of biomechanical movement performance in gait rehabilitation improve mobility outcomes after stroke?

### Secondary research questions

What impact will the visualisations have on patients’: understanding of their rehabilitation; motivation to achieve their rehabilitation goals; confidence in their ability to perform rehabilitation exercises; and adherence to rehabilitation?

## Methods

### Design

**Figure 3:**
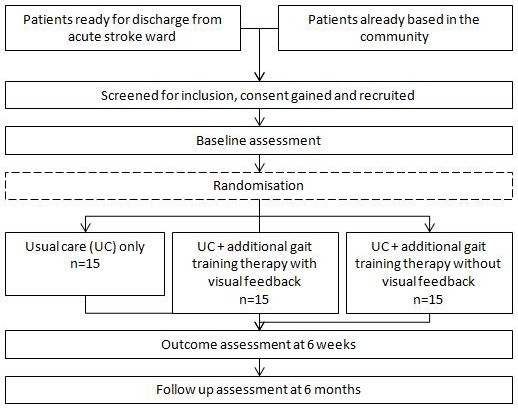
Outline of patient pathway in the randomised controlled trial.

The visualisation intervention will be tested in an exploratory (phase II) randomised controlled trial (RCT) [9]. See Figure3 for a flowchart of patient pathways. Participants will be randomised into three groups: a control group, a placebo group, and an intervention group that receives augmented visual feedback. 

Participants in the control group will continue with their usual care only. The term usual care refers to therapy provided by National Health Service (NHS) Lanarkshire, UK. This could be community care, day hospital care or no continued care, dependent on assessment of the patient’s ability by their clinical team.

In addition to usual care, participants in the placebo group will receive overground physical therapy gait training. Gait training will be provided as 1-hour sessions, twice weekly for 6 weeks. This training will consist of walking practice and a predetermined pool of walking-related exercises that study physiotherapists can choose from. The exercises are based on hip, knee and ankle range of motion in sitting and standing. Participants in this group will not receive any augmented visual feedback of their movement. The study physiotherapist will provide participants with feedback by demonstration and verbal feedback only, as per conventional gait training. During gait training, participants’ movements will be recorded using 3D optical motion capture. This will involve placing small reflective markers on the pelvis and lower limb segments.

In addition to usual care, participants in the intervention group will receive overground physical therapy gait training with augmented visual feedback of their movement performance. Gait training with visual feedback will be provided as 1-hour sessions, twice weekly for 6 weeks. During these gait training sessions, participants’ movements will be recorded using 3D optical motion capture, as in the placebo group, and will be used to power our bespoke visualisation software. In addition to feedback by demonstration and verbal feedback, participants in this group will receive augmented visual feedback in which they will be able to see a stick figure on a computer screen mimic their movements in real time. Sessions will consist of walking practice and the same predetermined pool of walking-related exercises as in the placebo group. However, each of the exercises will involve moving towards virtual targets. The virtual targets will be superimposed on the stick figure visualisation so that the patient is able to see how they move as they perform exercises. The study physiotherapist will have the option to provide virtual targets for movements of hip flexion/extension/abduction, knee flexion/extension, ankle dorsiflexion/plantarflexion and inversion/eversion. The study physiotherapist will have control over setting the ranges of motion for targets and the number of repetitions so that exercises can be tailored to an individual patient’s requirements. When to replay gait performances and view patient progress over time using the visualisations will be left to the study physiotherapist’s discretion.

The study physiotherapist will lead and be responsible for all clinical decision-making during placebo and intervention gait training sessions. The researcher will be present for technical support only. These sessions will be video-recorded for *post-hoc* analysis. Observational analysis of this data will allow comparison of patient–therapist interaction between intervention and placebo groups.

The primary outcome measure for this trial is walking speed, which will be measured using the 5-metre walk test [10]. Secondary outcome measures include the Functional Ambulation Category (FAC) [11], Timed Up and Go [12], Rivermead Visual Gait Assessment [13], spatial symmetry (ratio of step lengths) and temporal symmetry (ratio of step times), and the Stroke Impact Scale-16 [14]. Outcome assessments will be conducted at baseline, 6-week outcome and 6-month follow-up. The study physiotherapist will be present to assist participants during functional assessments only. The study researcher will be present to take measures and for technical support. To minimise patient burden during outcome assessments, participants will be instructed to do the 5-metre walk test four times and the Timed Up and Go test three times, if capable, while walking over a gridded mat and being video-recorded. From these recordings, an assessor blinded to treatment allocation will be able to assess all functional outcomes *post hoc*. The study researcher will help the participant complete the Stroke Impact Scale-16 questionnaire if required. Participants will be asked to complete a pre-trial questionnaire to determine what rehabilitation goals they may have and to obtain self-reported motivation and confidence levels. An additional questionnaire will be conducted by the study researcher at the 6-week outcome session. This will be used to gain further understanding of the impact and patient acceptance of the visualisation package. The questionnaire will explore whether the participant’s pre-trial goals have been achieved and the effects of the use of augmented visual feedback on patient motivation, confidence and understanding of their rehabilitation. The qualitative aspect of this research will be in the form of semi-structured interviews to gain further understanding of the impact of augmented visual feedback. These interviews will be carried out after participants have completed their 6-week outcome assessments. To reduce bias, an independent researcher, who has not been involved in the RCT, will be responsible for designing and conducting interviews with participants.

Impairments in the domains of attention and memory could be covariates in skill acquisition. Knowing the levels of impairment in these domains will enable us to check whether this has had an impact on the effectiveness of the intervention. The Rivermead Behavioural Memory Test [15], the Test of Everyday Attention [16] and the Line Cancellation Test [17] will therefore also be measured in the baseline session. Other outcomes will assess the safety of and patient adherence to the visualisation package. Safety will be assessed by the number and nature of adverse events throughout participation in the trial. Adherence will be assessed by the number of sessions attended and the withdrawal or dropout rate from the study. All therapy and outcome assessment sessions received in addition to usual care as part of the trial will take place at Coathill Day Hospital and Wishaw General Hospital, NHS Lanarkshire, UK.

### Identification of eligible patients

Participants will be recruited from acute sites and community rehabilitation teams in NHS Lanarkshire, UK. For full inclusion/exclusion criteria refer to Table 1. Potential participants will be identified by their NHS direct care team and/or stroke research nurses, who will ensure that the patient is able to give informed consent with suitable communication support if required. Written informed consent will be obtained from all participants prior to participation in the study.

**Table 1:** Inclusion and exclusion criteria

**Inclusion criteria**	**Exclusion criteria**
· Clinical diagnosis of stroke	· Severe visual or cognitive problems precluding participation in the study
· In the subacute stage of stroke (≤3 months since stroke onset)	· Involved in another physical rehabilitation research trial
· Of either gender	· Pre-existing lower limb deficits or any other medical comorbidities that interfere significantly with gait.
· Age ≥18 years	
· Exhibit an abnormal gait pattern Functional Ambulation Category of 1 or more	
· Medically stable and hence suitable for physical rehabilitation	
· Able to understand and follow simple instructions	
· Able to give informed consent when assisted to do so with suitable communication aids if required	

The research nurse or researcher will go over the study’s participant information sheet with the patient. Patients will have the opportunity to ask any questions and will be given a minimum of 24 hours to review the information sheet and consider their participation in the trial. During this time they will be able to ask any further questions and talk to other people about the study. The research nurse or researcher will then approach the patient again, answer any further questions and, if appropriate, gain consent from the patient who is to sign a consent form. This study complies with data-protection legislation. Upon enrolment in the study, patients will be allocated a unique trial number that will be used in all case-report file documents. Patient names will be used to arrange appointments only and will not be used for data-collection purposes. Where a patient is deemed eligible and provides informed consent, the following demographic and stroke information will be collected: age, sex, postcode, date of stroke, side affected, dominant side, pre-stroke function (FAC score) and type of stroke (Oxford Stroke Classification) [18].

### Data collection equipment

The 3D motion analysis will be carried out using an eight-camera Optitrack optical motion capture system (NaturalPoint, Corvallis, Oregon, USA). This will involve attaching small reflective markers on anatomical landmarks on the pelvis and lower limbs. The visualisation software will extract movement data from the motion capture system and present the data using bespoke visualisations.

### Sample size

A GPower *a priori* analysis for a mixed (2 × 3) analysis of variance was carried out. Setting α at 0.05 and 1β at 0.8, with an effect size of 0.2, this predicted a total sample size of 42. On this basis, and accounting for roughly a 10% drop out, we estimated that 45 participants (15 per group) would be sufficient to minimise the risk of type 1 or type 2 errors. We anticipate that results from this pilot study will yield data for a sample size estimation for a future definitive RCT.

### Randomisation method

The randomisation sequence will be generated using an independently verified S-PLUS program (Robertson Centre for Biostatistics, University of Glasgow, UK). This program uses a stratified permuted block randomisation procedure, with stratification based on the severity of walking impairment. This stratification will ensure that intervention groups are balanced, since severity of impairment influences the potential for recovery. Severity of walking impairments will be classified using the FAC. Stratification will consist of two groups: FAC 1 to 3, and FAC 4 to 5. The researcher will access intervention allocations through the study web portal, designed and maintained by The Robertson Centre for Biostatistics at the University of Glasgow. The randomisation sequence will be concealed until the participant’s details are logged on the system.

### Statistical analysis

Data will be analysed on an intention to treat basis. Data will be held independently on a database at the Glasgow Clinical Trials Unit at the University of Glasgow. Datasets for each randomisation allocation will be compared by independent blinded assessors.

Descriptive statistics will be calculated and the characteristics of the population will be described. Two mixed-design analysis of variance models will be used to test for mean differences between the three arms of the RCT, while subjecting participants to repeated measures. The repeated measures will be examined for differences over time and between groups. If appropriate, factors that may affect the outcome of the trial – such as differences in time since onset of stroke, age, severity of stroke or memory and attention levels – will be investigated using demographic and stroke-related information collected at baseline.

Data collected from pre-trial and post-trial interviews by the study researcher and in-depth semi-structured interviews by an independent researcher will be suitably coded such that qualitative responses can be categorised and explored.

## Discussion

This protocol describes a phase II (or exploratory) RCT that aims to explore the effect of using visual feedback of biomechanical movement performance as an adjunct to gait training after stroke. Stroke survivors and stroke rehabilitation professionals, clinicians and academics, have been involved in both the development of this protocol and the visualisation software. Our findings from informal semi-structured interviews and focus groups have suggested that dynamic visualisations could be a useful addition to gait rehabilitation after a stroke. Involving users in the development of the software has meant visualisations have been designed to specifically complement lower-limb stroke rehabilitation.

Therapy and outcome sessions could possibly be influenced by the knowledge and experience of individual therapists. To reduce this variability, one physiotherapist will lead all trial therapy sessions that are provided in addition to the patient’s usual care.

The trial will focus on quantitative functional outcomes; however, it will incorporate qualitative measures, including patient-reported questionnaires, to aid in the explanation of outcomes. Qualitative data capture will be analysed to provide clarity on the impact of the visualisations with regards to participant confidence, motivation and understanding of their rehabilitation programme as a result of taking part in the trial. This single-site study, although relatively small scale, is expected to provide valuable evidence to support the early mobilisation of stroke survivors through visualisation of movement performance. We anticipate that results will confirm benefits associated with using visualisation of biomechanical data during gait training after stroke, and will be used to power a future larger-scaled study.

Furthermore, the RCT aims to address whether the visualisations will engage clients in their rehabilitation, enabling them to perform to their optimal functional capability. This method retains the accuracy and accessibility of the biomechanical data required by clinicians but presents the data in a nontechnical way, facilitating enhanced communication between patient and physiotherapist and patient understanding of their rehabilitation. Patients will be able to see how they move and will be able to observe any compensatory strategies they may employ to complete rehabilitation tasks. This application has the potential to enable patients to self-correct their movement patterns and discourage overreliance on their physiotherapists for corrective advice. Moreover, such a stroke rehabilitation tool could provide the objective training required for assessment during rehabilitation. Tracking progress after a stroke can be difficult as improvements can be small and can take place over long periods of time. Retaining movement data over the rehabilitation programme enables one to compare different therapy sessions. For example, a physiotherapist could visually view progress between baseline and consequent sessions by pulling up saved files that display stick figure visualisations mimicking how the user moved in those particular sessions. This visualisation could instantly allow the physiotherapist to get an idea of the quality of the patient’s movements, and supporting numerical data could also be accessed to compare ranges of motion achieved over time.

### Trial status

Ethical approval was obtained from NHS West of Scotland Research Ethics Committee 2 on 26 April 2011 (Ref 11/AL/0184). NHS Lanarkshire R&D approval was obtained on 5 April 2011. The trial commenced in March 2012.

## Abbreviations

3D : three-dimensional; FAC : Functional Ambulation Category; NHS : National Health Service.

## Competing interests

The authors declare that they have no competing interests.

## Authors’ contributions

HT developed the study protocol and drafted and revised the manuscript. MG and FvW helped to design the study and draft the manuscript. MB and PR played significant roles in the design and coordination of the study and also helped to draft the manuscript. All authors read and approved the final manuscript.
